# Definition of giant inguinoscrotal hernias in infants and evaluation of reliable surgical approach in a single-center study

**DOI:** 10.1016/j.amsu.2019.07.002

**Published:** 2019-07-09

**Authors:** Ali İhsan Anadolulu, Mehmet Tolga Kafadar, Gonca Gerçel

**Affiliations:** a Health Sciences University Mehmet Akif İnan Training and Research Hospital Clinic of Pediatric Surgery, Şanlıurfa, Turkey; b Health Sciences University Mehmet Akif İnan Training and Research Hospital Clinic of General Surgery, Şanlıurfa, Turkey; c Şanlıurfa Training and Research Hospital Clinic of Pediatric Surgery, Şanlıurfa, Turkey

**Keywords:** Inguinoscrotal hernia, Infant, Zig maneuver

## Abstract

**Objective:**

Inguinal hernia surgery is the most common surgery performed by pediatric surgeons. Giant inguinoscrotal hernia has not been clearly defined yet. The definition of giant inguinoscrotal hernia and the reliability of the surgical procedure were investigated in this study.

**Materials and methods:**

Sixtyfour of totally 1548 male patients who have been operated with inguinal hernia from May 2015 to January 2018 were included in the study considering the diagnosis of giant inguinoscrotal hernia. The criteria for the diagnosis of giant inguinoscrotal hernia were determined as, observing that the hernia sac was filled with intestinal loops from the inguinal region to the scrotum during the physical examination, herniation of the intestines to the scrotum again as soon as the hernia was reduced and 2 cm and above inner ring diameter. High ligation and hernioplasty to 29 (45.3%) patients and hernioplasty using Zig maneuver to 35 (54.6%) patients were performed during the study.

**Results:**

Postoperative wound infection was observed in 2 patients (6.8%) with high ligation and 1 (2.8%) patient with hernioplasty with Zig maneuver. Scrotal edema was detected in all the patients, which persisted until postoperative month 1. Recurrence was seen in 6 (20.6%) of 29 patients who operated using the high ligation method while it was seen in 2 (5.7%) of other 35 patients. None of the patients had testicular atrophy and/or iatrogenic undescended testis.

**Conclusion:**

Giant inguinoscrotal hernias should be defined and evaluated as a group apart from classical inguinoscrotal hernias. Recurrence and morbidity rates were lower in patients who underwent hernioplasty using Zig maneuver.

## Highlights

• The definition of giant inguinoscrotal hernia and the reliability of the surgical procedure were investigated clearly in this study.
• Giant inguinoscrotal hernias should be defined and evaluated as a group apart from classical inguinoscrotal hernias.
• Giant inguinal hernias are rare in children and the treatment require surgical experience.

## 1 Introduction

Even though herniation of the intestines from the inguinal canal to the scrotum is considered inguinoscrotal hernia, the giant inguinoscrotal hernia has not been clearly defined yet in the literature. The incidence of inguinal hernia in term male infants is 3% and this rate can be as high as 30% in preterms. Surgery is the only treatment today for inguinal hernia. The preferred surgical technique is high ligation. In large series, recurrence rate in high ligation has been reported below 2%. Recurrence rate in giant inguinoscrotal hernia is around 10%. Dissection of the sac from the cord and vasculatures is very difficult and the probability of rupture of the sac is high and requires a surgical experience. Turning processus vaginalis with Zig maneuver is a very reliable method for protecting vas deferens and vascular structures without disrupting the integrity of the sac [1]. The aim of the present study was to investigate the definition of giant inguinoscrotal hernia and the recurrence rate in patients with and without Zig maneuver.

## 2 Material and methods

**Fig. 1:**
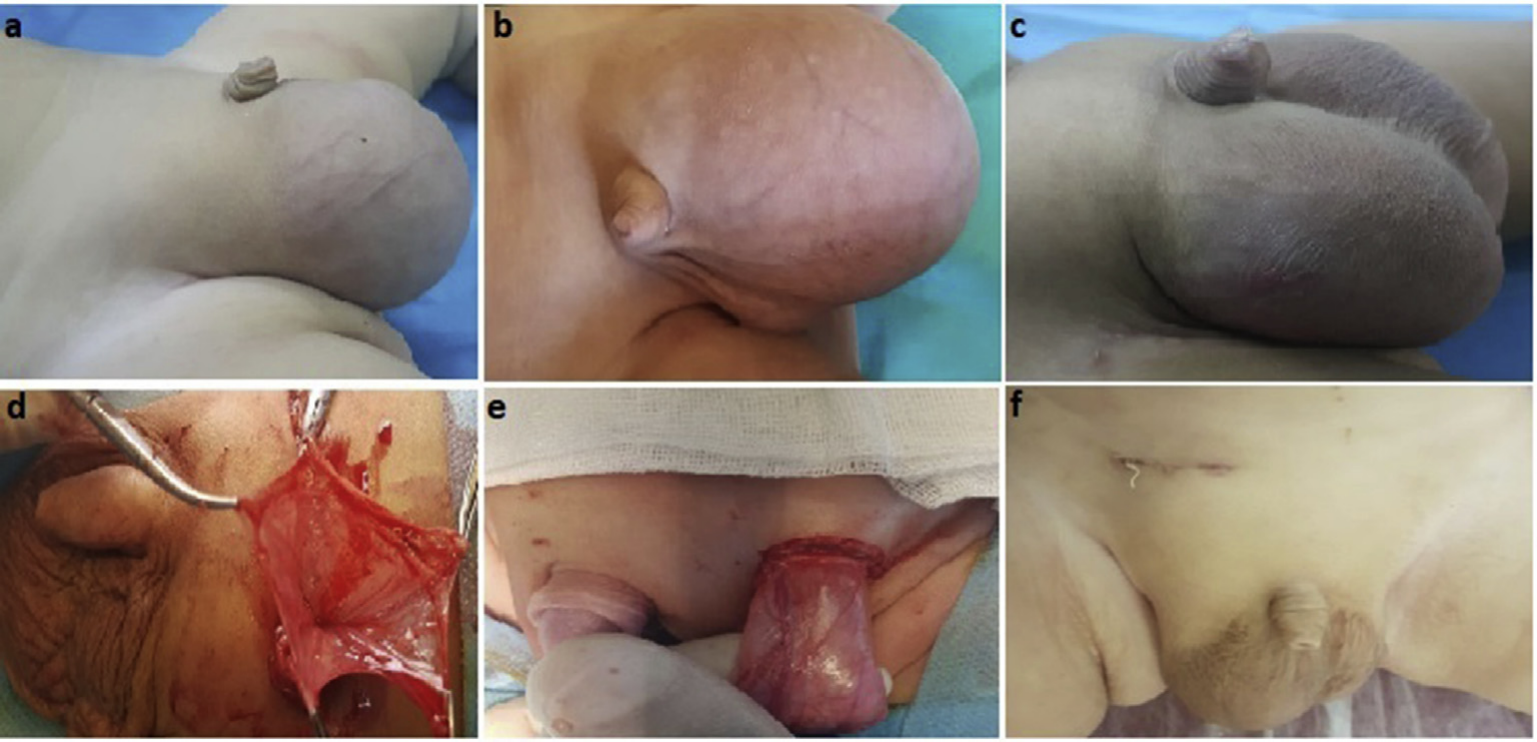
Preoperative view of the patients (a–c), intraoperative view of the patients (d,e), postoperative view of the patient (f).

Sixtyfour (4%) of totally 1548 male patients (1389 unilateral, 159 bilateral) who have been operated with inguinal hernia from May 2015 to January 2018 were included in the study considering the diagnosis of giant inguinoscrotal hernia. The criteria for the diagnosis of giant inguinoscrotal hernia were determined as, observing that the hernia sac was filled with intestinal loops from the inguinal region to the scrotum during the physical examination, herniation of the intestines to the scrotum again as soon as the hernia was reduced and 2 cm and above inner ring diameter (Fig. 1a–c). All operations were performed by a single surgeon under general anesthesia. Of the 64 patients with giant inguinoschrotal hernia, 8 were bilateral and 56 were unilateral. Bilateral hernias were operated simultaneously. High ligation hernioplasty was performed for the first 29 (45%) patients included in the study while hernioplasty with Zig maneuver was performed for the other 35 (55%) patients due to high recurrence rate (Fig. 1d–f). During the Zig maneuver, after crossing the groin layers in a standard way, hernia sac was grasped from anterior side taking a large gentle bite of tissue. Then the hernia sac was opened from the anterior side voluntarily without attempting to dissect it from the vas deferens and testicular vessels. After the four walls of the sac were suspended by clamps, the vas deferens and vessels adjacent to the inferior wall were dissected from the sac wall by checking them inside of the sac wall as well as the outside. Until the integrity of the sac walls was checked and the preperitoneal adipose tissue was seen, the sac was separated from the surrounding tissues. The sac was then cleaned to the level of the internal ring, twisted on itself and ligated at the level of the internal ring with using 2 pieces of no. 3-0 absorbable suture. At the end internal inguinal ring was narrowed externally with absorbable sutures in all patients.

## 3 Results

**Fig. 2:**
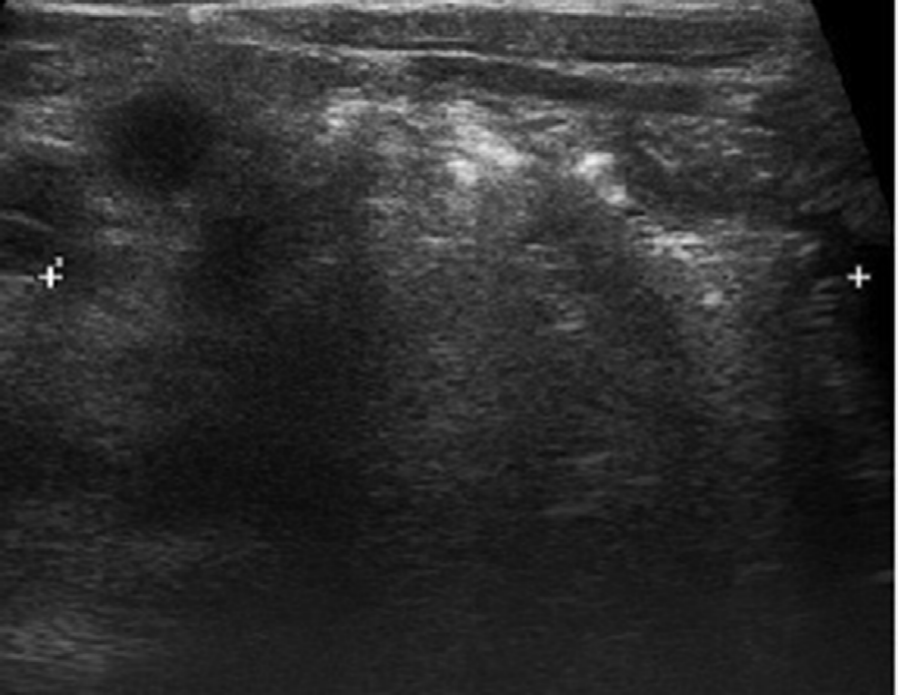
Ultrasonografic view of inguinal hernia sac.

The median age of the 64 patients included in the study was 9 (6–48) months. Physical examination revealed post-reductive hernias that re-fill the scrotum quickly. No incarceration was observed in any patient. During ultrasonographic imaging, diameter of internal ring was found 2 cm and above (2–4.8) (Fig. 2). They had no comorbidities. Postoperative wound infection was observed in 2 patients with high ligation and 1 patient with hernioplasty with Zig maneuver. Patients with emerged infection had positive response to the medical treatment. Scrotal edema was detected in all the patients, which persisted until postoperative month 1. Recurrence was seen in 6 (20.6%) of 29 patients who operated using the high ligation method while it was seen in 2 (5.7%) of other 35 patients. The mean follow-up period was 20 (min: 2, max: 36) months. None of the patients had testicular atrophy and/or iatrogenic undescended testis.

## 4 Discussion

Indirect inguinal hernias in children are caused by remaining of the processus vaginalis open. The extension of the intraabdominal organs from this opening to the scrotum is defined as inguinoscrotal hernia. Inguinoscrotal hernias are more common in adulthood and occur secondary to long-term herniation. It is very rare other than the presence of predisposing factors such as ventriculoperitoneal shunt and ascites in childhood. Giant hernias in the inguinoscrotal hernia group have been reported mostly as case reports in the literature [2]. However, there is no clear diagnostic criterion for giant inguinal hernia. In giant hernia, internal inguinal ring is wider than inguinoscrotal hernia. Dilated internal inguinal canal inlet does not allow permanent reduction of intestines. This may require perioperative additional procedures [3]. Therefore, we think that this group needs a definition apart from inguinoscrotal hernias. For this purpose, we tried to establish the diagnostic criteria for giant hernias in our study. The criteria for the diagnosis of giant inguinoscrotal hernia were determined as, observing that the hernia sac was filled with intestinal loops from the inguinal region to the scrotum during the physical examination, herniation of the intestines to the scrotum again as soon as the hernia was reduced and 2 cm and above inner ring diameter.

The fundamental principle guiding pediatric inguinal hernia repair is high ligation of the hernia sac. Since the intestines are in the scrotum as of the neonatal period, the hernia sac is conjoined to the surrounding tissues and fragile. Inguinal canal fascia gained flexibility secondary to compression. The thickening of the creamaster muscles makes the dissection of the sac from the cord and veins difficult, which is performed during standard high ligation. In giant hernias, it is much more likely to damage these structures when trying to separate the sac from the cord and testicular vessels [4]. In hernioplasty with Zig maneuver, the vas deferens and vessels adjacent to the sac wall could be dissected from the sac wall meticulously by checking them inside of the sac wall as well as the outside. Because of the thickened tissues in giant hernias, it can be very difficult to distinguish the cord and veins from the surrounding tissues. Therefore, we could say that hernioplasty with Zig maneuver is safer method than standard high ligation hernioplasty.

The recurrence rate has been reported 10% in the literature for operation of inguinoscrotal hernias via hernioplasty with high ligation. Recurrence rates higher than standard inguinal hernias led to the look for different surgical techniques in this group of patients [5]. Banieghbal has specified in his series, that performing dissection after taking out testis out of the inguinal incision reduced the duration of operation and regressed the recurrence rate [6].

Hill and Durham specified that they used a biological mesh for a neonatal case with giant hernia and that using a mesh strengthen the muscle layer and thus prevented recurrence [7]. No mesh was used for any patient in our study. Contrary to repair using a mesh used commonly in adults, more flexible tissues in the childhood age group make it possible to perform primary repair without creating tension in tissues.

Recurrence rates were compared in our study after hernioplasty with high ligation and zig maneuver in infants. Performing hernia surgery in this category via standard hernioplasty can increase the rate of recurrence as revealed in our study [8]. High ligation hernioplasty was performed for the first 29 patients in this series while hernioplasty with zig maneuver was performed for the other 35 patients due to high recurrence rate. Internal inguinal ring was narrowed in all patients.

In hernioplasty with Zig maneuver, the sac walls are first suspended and less traction and dissection is applied to the cord and testicular vessels. Most of surgeons does not check the wall integrity by opening the sac after dissecting it in the high ligation. In giant hernias where the sac walls are more fragile and attached to the surrounding creamaster muscles, matters regarding perforation in proximal segment to the sac or the fact whether the four walls of the sac are dissected or not can easily be overlooked. In our preferred method, since the sac is suspended via clamps as of the beginning of the operation with clamps and the inner surface of the sac is visible, the integrity of the sac is in sight and this reduces the possible recurrences [9]. In current study, when the recurrence rates between the two groups were evaluated, the rates were found lower in the hernioplasty with Zig maneuver group.

Narrowing of the inner ring using primary sutures that is another procedure we recommend for giant hernias, forms a supporting tissue at the base of the inguinal canal. It prevents subcutaneous palpation of the hernia sac stump and evaluation of swelling in the inguinal region as recurrence by mistake during the effort movements especially in children with less weight [10]. Yokomori et al. have narrowed the internal inguinal canal by a modified Marcy repair and reported in their series that this method was effective to prevent recurrence [11]. In our study internal inguinal ring was narrowed in all patients.

In conclusion, giant inguinal hernias are rare in children and the treatment require surgical experience. These types of hernias should be defined and evaluated as a group apart from classical inguinoscrotal hernias. Recurrence and co-morbidity can be decreased in giant hernias through the surgical technique specified in our study.

## Ethical approval

Authors declared that the research was conducted according to the principles of the World Medical Association Declaration of Helsinki “Ethical Principles for Medical Research Involving Human Subjects”, (amended in October 2013).

## Sources of funding

The authors declared that this study has received no financial support

## Author contributions

Concept – A.İ.A., M.T.K.; Design - M.T.K.; Supervision- A.İ.A.; Resources - M.T.K., G.G.; Materials – A.İ.A., G.G., Data Collection and/or Processing – A.İ.A., G.G.; Analysis and/or Interpretation - M.T.K.; Literature Search – M.T.K., G.G.; Writing Manuscript – A.İ.A., M.T.K.; Critical Review - M.T.K.

## Conflicts of interest

No conflict of interest was declared by the authors.

## Trial registry number

Unique Identifying Number (UIN): 4972.

## Guarantor

Mehmet Tolga Kafadar.

## Consent

Written informed consent was obtained from the fathers of the patients for publication of this article and accompanying images. A copy of the written consent is available for review by the Editor-in-Chief of this journal on request.

## Annals of medicine and surgery

The following information is required for submission. Please note that failure to respond to these questions/statements will mean your submission will be returned. If you have nothing to declare in any of these categories then this should be stated.
